# *Niabella beijingensis* sp. nov. and *Thermomonas beijingensis* sp. nov., two bacteria from constructed wetland

**DOI:** 10.1099/ijsem.0.005280

**Published:** 2022-03-22

**Authors:** Sheng-Zhi Guo, Tong Wu, Hai-Zhen Zhu, Lei Yan, Zhi-Pei Liu, De-Feng Li, Cheng-Ying Jiang, Shuang-Jiang Liu, Xi-Hui Shen

**Affiliations:** ^1^​ State Key Laboratory of Crop Stress Biology for Arid Areas, Shaanxi Key Laboratory of Agricultural and Environmental Microbiology, College of Life Sciences, Northwest A&F University, Yangling, Shaanxi 712100, PR China; ^2^​ State Key Laboratory of Microbial Resources and Environmental Microbiology Research Center at Institute of Microbiology, Chinese Academy of Sciences, Beijing 100101, PR China; ^3^​ University of Chinese Academy of Sciences, Beijing 100049, PR China; ^4^​ State Key Laboratory of Microbial Biotechnology, Shandong University, Qingdao 266237, PR China

**Keywords:** *Niabella beijingensis *sp. nov., *Thermomonas beijingensis *sp. nov., strain RSS-23^T^, strain 3A5MI-3^T^, CGMCC 1.17737^T^=KCTC 82817^T^, CGMCC 1.17738^T^=KCTC 82820^T^

## Abstract

Two Gram-stain-negative, aerobic, non-motile and rod-shaped bacterial strains designated 3A5MI-3^T^ and RSS-23^T^ were isolated from the Dragon-shaped Wetland System in Beijing Olympic Park, PR China. Strain 3A5MI-3^T^ grew at 15–45 °C, pH 5.0–9.0 and with 0–2 % NaCl (w/v), and strain RSS-23^T^ grew at 15-40 ^o^C, pH 5.5–9.0 and with 0–1 % NaCl (w/v). Phylogenetic analyses of 16S rRNA gene sequences revealed that strains 3A5MI-3^T^ and RSS-23^T^ were members of *Bacteroidetes* and *Proteobacteria *, respectively. Phylogenetically closest relatives of strains 3A5MI-3^T^ and RSS-23^T^ were *Niabella pedocola* R384^T^ and *Thermomonas aquatica* SY21^T^, respectively. The cells of strain 3A5MI-3^T^ contained menaquinone MK-7 and phosphatidylethanolamine, and the major cellular fatty acids were composed of iso-C_15 : 0_, iso-C_15 : 1_ ω6*c* and/or iso-C_15 : 1_ ω7*c*, iso-C_17 : 0_ 3-OH, C_16 : 0_ and summed feature 3 (C_16 : 1_ ω7*c*/C_16 : 1_ ω6*c*). Strain RSS-23^T^ contained ubiquinone Q-8 and diphosphatidylglycerol, phosphatidylglycerol, phosphatidylethanolamine, two unknown phospholipids and an unknown lipid, and its major cellular fatty acids were iso-C_15 : 0_, iso-C_17 : 1_ ω9*c*, iso-C_11 : 0_ 3-OH and summed feature 3 (C_16 : 1_ ω7*c*/C_16 : 1_ ω6*c*). DNA sequencing resulted in 6.59 Mb for the strain 3A5MI-3^T^ genome and 2.79 Mb for the strain RSS-23^T^ genome. The calculated G+C molar contents for strains 3A5MI-3^T^ and RSS-23^T^ were 47.07 and 61.21 mol%, respectively. According to phenotypic and phylogenetic characteristics, strains 3A5MI-3^T^ and RSS-23^T^ represent novel species of the genera *Niabella* and *Thermomonas* for which the names *Niabella beijingensis* sp. nov. and *Thermomonas beijingensis* sp. nov. are proposed. The type strain for *N. beijingensis* sp. nov. is 3A5MI-3^T^ (=CGMCC 1.17737^T^=KCTC 82817^T^). The type strain for *T. beijingensis* sp. nov. is RSS-23^T^ (=CGMCC 1.17738^T^=KCTC 82820^T^).

## Data Summary

The newly sequenced data included in this work are deposited under the nucleotide accession numbers: MZ919349 and MZ920050 and under the Bioproject accession numbers JAIQDI000000000, JAJNEC000000000 and JAIQDJ000000000 at a public domain server in the National Center for Biotechnology Information (NCBI) database.

All supporting data, code and protocols have been provided within the article or through supplementary data files. A supplementary table and further supplementary files can be found at: https://doi.org/10.6084/m9.figshare.18220907.v1 [1].

Although members of the genera *Niabella* and *Thermomonas* are often isolated from similar environmental samples [2–5], they belong to the *Bacteroidetes* and *Proteobacteria *, respectively. The genus *Niabella* is a member of the family *Chitinophagaceae *. Cells of *Niabella* species are Gram-stain-negative, aerobic, non-flagellated and short rods. Since the genus *Niabella* was firstly described by Kim *et al*. [6], 11 specific names have been validly published [2, 3, 6–13] (https://lpsn.dsmz.de/genus/niabella). The names of ‘* Niabella terrae *’ [14] and ‘* Niabella thaonhiensis *’ [15] were proposed but have not been validated at the time of this writing. The genus *Thermomonas *, a member of the family *Xanthomonadaceae *, was firstly described by Busse *et al*. [16]. At the time of writing, seven species have been validly published [4, 5, 16–19] (https://lpsn.dsmz.de/genus/thermomonas). The species of the genera *Niabella* and *Thermomonas* have been isolated from a wide range of habitats, and they share features such as cells that are Gram-strain-negative, aerobic and non-flagellated.

In this report, we describe two bacterial strains that were isolated from a constructed wetland system, by phylogenetic, physiological, biochemical, and genomic analyses.

The constructed wetland system, also called the Dragon-shaped Wetland, located in the central area of the Beijing Olympic Park area (40° 0.2′ N and 116° 22.28′ E), is the largest urban artificial water system in Asia. The water in the Dragon-shaped Water System comes mainly from the Beijing Qinghe Water Reclamation Plant, and the entire water system has a complete circulation system. Sludge samples from the Dragon-shaped Wetland water system were collected, and were serially diluted with 0.85 % NaCl (w/v) and plated onto Reasoner's 2A (R2A) agar (Difco). The isolates, designated as 3A5MI-3^T^ and RSS-23^T^, were obtained after incubation for 3 days at 30 °C and were routinely stored at −80 °C as suspensions in R2A broth supplemented with 20 % (v/v) glycerol.

Genomic DNA was extracted with a commercial TIANamp Bacteria DNA Kit, and 16S rRNA genes were amplified by PCR with universal bacterial primers 27 F and 1492R [20], which was also used for sequencing the PCR product. The almost-complete sequence was compared with 16S rRNA gene sequences from GenBank. The 16S rRNA gene sequences were aligned using ClustalW [21]. Phylogenetic analyses were carried out using three phylogenetic algorithms: neighbour-joining [22], maximum-likelihood [23] and maximum-parsimony [24]. Phylogenetic trees were reconstructed and bootstrapped with 1000 replicates of each sequence using mega version 7.0 [25]. The CVTree method was used to reconstruct phylogenomic tree based on whole genomes [26].

**Fig. 1.**
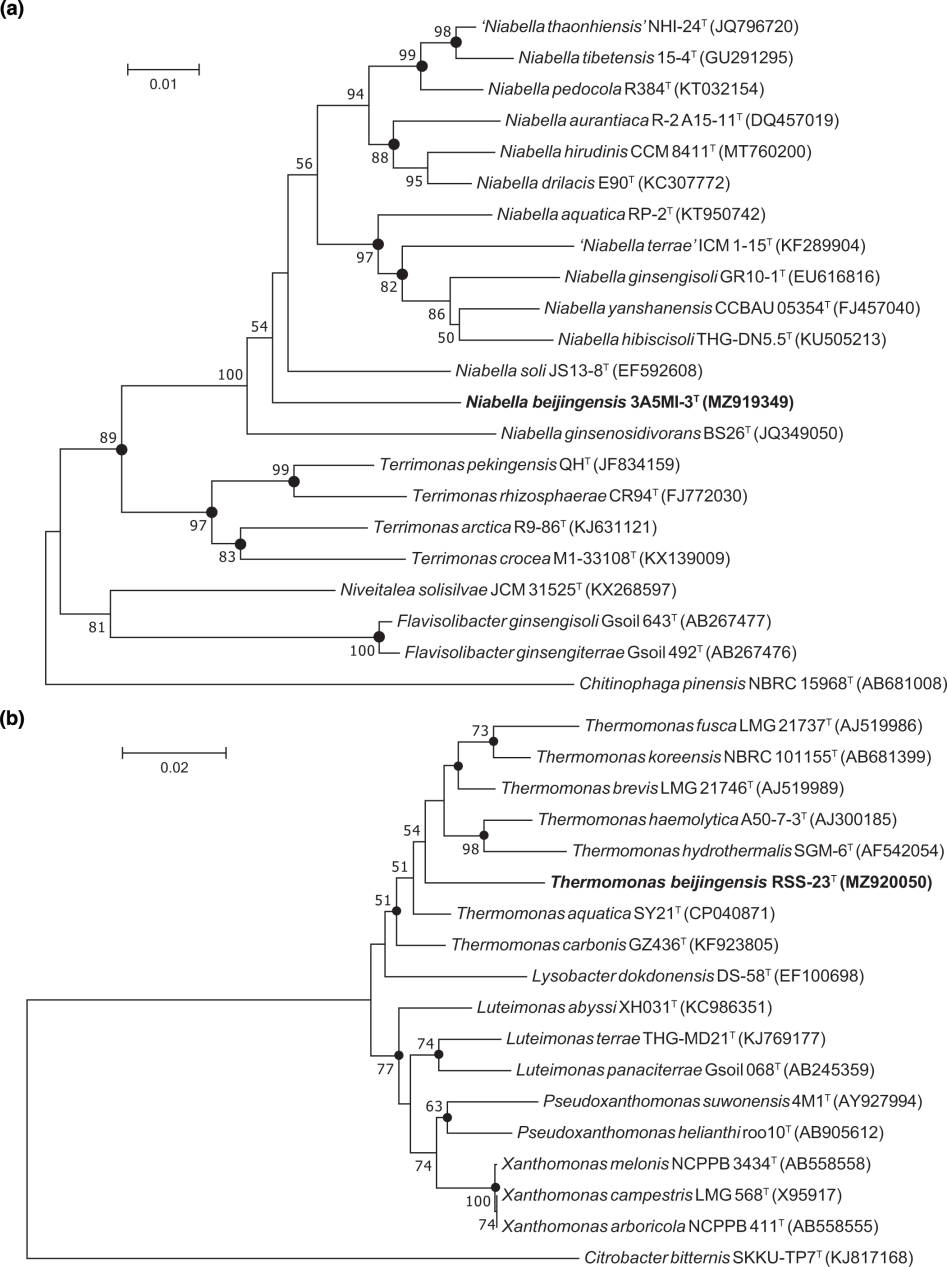
Neighbour-joining phylogenetic tree based on 16S rRNA gene sequences, showing the phylogenetic position of strains 3A5MI-3^T^ (**a**) and RSS-23^T^ (**b**) among closely related taxa. GenBank accession numbers are given in parentheses. Numbers at nodes indicate the percentage of 1000 bootstrap replicates yielding this topology; only values >50 % are shown. Filled circles indicate nodes recovered by all three treeing methods (neighbour-joining, maximum-likelihood and maximum-parsimony). *Chitinophaga pinensis* NBRC 18968^T^ and *Citrobacter bitternis* SKKU-TP7^T^ were used as outgroups.

Analysis of 16S rRNA gene sequences in GenBank indicated that strain 3A5MI-3^T^ was phylogenetically close to *Niabella pedocola* R384^T^ (95.97 %), ‘* Niabella thaonhiensis *’ NHI-24^T^ (95.68 %), *Niabella aurantiaca* R2A15-11^T^ (95.61 %), *Niabella tibetensis* 15-4^T^ (95.60 %), *Niabella soli* JS 13-8^T^ (95.25 %), *Niabella drilacis* E90^T^ (94.62 %), *Niabella hirudinis* CCM8411^T^ (94.53 %), *Niabella aquatica* RP-2^T^ (93.93 %), *Niabella yanshanensis* CCBAU 05354^T^ (93.76 %), *Niabella ginsenosidivorans* BS26^T^ (93.51%), ‘* Niabella terrae *’ ICM 1-15^T^ (92.66 %), *Niabella hibiscisoli* THG-DN5.5^T^ (92.52 %) and *Niabella ginsengisoli* GR 10-1^T^ (92.45 %), as well as to *Terrimonas rhizosphaerae* CR94^T^ (92.14 %) and was <92 % similar to some other species included in this analysis (Fig. 1a and S1, available in the online version of this article). Based on the results of phylogenetic analysis and 16S rRNA gene identity, *N. pedocola* JCM 31011^T^ was selected as a reference strain for phenotypic tests (see following paragraph).

**Fig. 2.**
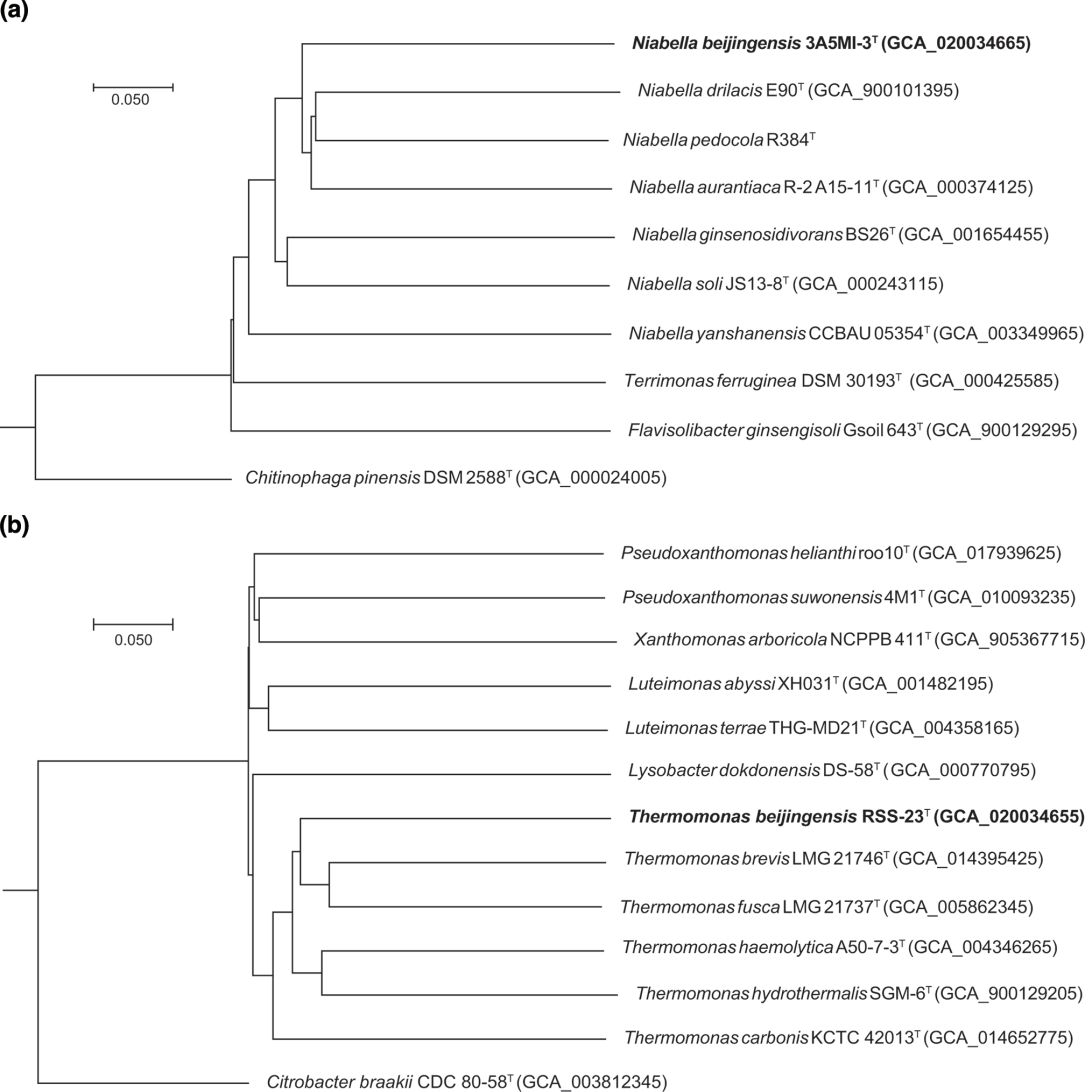
Phylogenomic trees of strains 3A5MI-3^T^ (**a**) and RSS-23^T^ (**b**). GenBank accession numbers are given in parentheses. We performed genome sequencing of *Niabella pedocola* JCM31011^T^ and the sequence was deposited in the GenBank/EMBL/DDBJ with the accession number JAJNEC000000000. *Chitinophaga pinensis* DSM 2588^T^ (=NBRC 18968^T^) and *Citrobacter braakii* CDC 80-58^T^ (=SKKU-TP7^T^) were used as outgroups.

Strain RSS-23^T^ was phylogenetically close to *Thermomonas aquatica* SY21^T^ (96.99 %)*, Thermomonas fusca* LMG 21737^T^ (96.40 %), *Thermomonas brevis* LMG 21746^T^ (95.90 %), *Thermomonas carbonis* GZ436^T^ (95.82 %), *Thermomonas haemolytica* A50-7-3^T^ (95.75 %), *Thermomonas koreensis* NBRC 101155^T^ (95.61 %) and *Thermomonas hydrothermalis* SGM-6^T^ (95.44 %) (16S rRNA gene sequence identities >95 %). The phylogenetic tree showed that the strain grouped with seven members of the genus *Thermomonas* (Fig. 1b and S2). A phylogenomic tree based on genome sequences also showed the phylogenetic positions of strains 3A5MI-3^T^ (Fig. 2a) and RSS-23^T^ (Fig. 2b).

To analyse the genomic properties of strains 3A5MI-3^T^ and RSS-23^T^, whole-genome sequencing was performed using the Illumina system. The genomic assembly was performed with the SPAdes software (version 3.9.0) using clean data [27]. Average nucleotide identity (ANI) values were calculated with ChunLab’s online ANI Calculator [28]. Digital DNA–DNA hybridization (dDDH) was performed by using the Genome-to-Genome Distance Calculator (3.0) [29].

The genome of strain 3A5MI-3^T^ contained three contigs, and the total length was 6.59 Mb, encoding 6358 genes. The calculated DNA G+C content of strain 3A5MI-3^T^ was 47.07 mol%. The genome of strain 3A5MI-3^T^ carries 49 ncRNA genes, including three rRNA operons (5S, 16S, 23S), 41 tRNA genes and five sRNA genes. In addition, there is a clustered regularly interspaced short palindromic repeat sequence (CRISPR) with a length of 2862 bp in this genome. The Kyoto Encyclopedia of Genes and Genomes (KEGG) functional category distribution revealed that large numbers of genes in the genome of strain 3A5MI-3^T^ belonged to the metabolism (61 %), genetic information processing (16 %), environmental information processing (7 %) and cellular processes (7 %). Annotated with the RefSeq non-redundant proteins (NR) database, the genomes of strain 3A5MI-3^T^ harboured nine genes encoding phosphohydrolase, nine genes encoding phosphatase, 11 genes encoding glucosidase, 51 genes encoding glycosyl hydrolase families, 36 genes encoding starch binding protein, six genes encoding polysaccharide lyase, seven genes encoding polysaccharide deacetylase, 28 genes encoding SusC/RagA family TonB-linked outer membrane protein, 13 genes encoding xylanase, one gene encoding cellulase and one gene encoding chondroitinase-B. We found genes for xylanase and cellulase in the strain 3A5MI-3^T^ and *N. pedocola* JCM31011^T^ genomes. APIZYM and carbon source utilization experiments confirmed that strain 3A5MI-3^T^ and *N. pedocola* JCM31011^T^ could utilize xylan and cellulose. Although strain 3A5MI-3^T^ and *N. pedocola* JCM31011^T^ both have the chitinase gene in their genome, neither of them can use chitin. Trypsin-like serine proteases were found in the strain 3A5MI-3^T^ genome but not in strain *N. pedocola* JCM31011^T^, which corresponds to APIZYM trypsin activity for strain 3A5MI-3^T^ but not for *N. pedocola* JCM31011^T^. Comparing the genomes of strain 3A5MI-3^T^ and *N. pedocola* JCM 31011^T^, we found that the specific genes detected in strain 3A5MI-3^T^ were as follows: four genes encoding lipopolysaccharide biosynthesis protein, three genes encoding biotin synthase, three genes encoding CRISPR-associated protein Cas1, two genes encoding 2-dehydropantoate 2-reductase, one gene encoding bifunctional NAD(P)H-nitrite reductase/anaerobic dehydrogenase, two genes encoding arabinogalactan endo-1,4-beta-galactosidase, three genes encoding TDP-4-oxo-6-deoxy-d-glucose aminotransferase, one gene encoding zinc-binding alcohol dehydrogenase family protein, three genes encoding 3-phytase, three genes encoding alpha-glucuronidase and one gene encoding Alg9-like mannosyltransferase family protein. The accession numbers of the sequences in the database relating to the specific or characteristic (marked in yellow) genes of strain 3A5MI-3^T^ and *N. pedocola* JCM 31011^T^ are provided as supplementary material. The ANI value between strains 3A5MI-3^T^ and *N. pedocola* JCM 31011^T^ was calculated using ChunLab’s online ANI Calculator [28]. We performed genome sequencing of *N. pedocola* JCM31011^T^ and its GenBank/EMBL/DDBJ accession number is JAJNEC000000000. The ANI result was 77.48 %, below the 95 % inter-species threshold [30]. dDDH was performed with genome sequences of strains 3A5MI-3^T^ and *N. pedocola* JCM 31011^T^ using the Genome-to-Genome Distance Calculator 29]. The estimated dDDH value was 19.50 %, which was below the standard value generally recommended for species differentiation.

The genome of strain RSS-23^T^ contained also three contigs, and the total length is 2.79 Mb, encoding 2575 genes. The calculated genomic DNA G+C content of strain RSS-23^T^ was 61.21 mol%. The genome of strain RSS-23^T^ carries 58 ncRNA genes, including six rRNA genes (5S, 16S, 23S), 49 tRNA genes and three sRNA genes. The KEGG functional category distribution revealed that large numbers of genes in the genome of strain RSS-23^T^ belonged to the metabolism (53 %), genetic information processing (16 %), environmental information processing (14 %), cellular processes (8 %). Annotated with the RefSeq NR database, the genomes of strain RSS-23^T^ harboured 61 genes encoding phosphatase, six genes encoding glucosidase, four genes encoding glycosyl hydrolase, 27 genes encoding glycosyl transferase and 11 genes encoding polysaccharide biosynthesis protein. APIZYM positive enzyme genes such as alkaline phosphatase, acid phosphatase and α-glucosidase were also found in the genome. Comparing the genomes of strains RSS-23^T^ and *T. fusca* DSM 15424^T^, we found that the specific genes contained in strain RSS-23^T^ are as follows: three genes encoding aminotransferase class I/II, six genes encoding chloride channel protein, three genes encoding penicillin acylase, three genes encoding UDP-glucose 4-epimerase, three genes encoding UDP-N-acetylglucosamine 2-epimerase and three genes encoding carbonate dehydratase. The accession numbers of the sequences on the database relating to the specific or characteristic (marked in yellow) genes of strain RSS-23^T^ and *T. fusca* DSM 15424^T^ are provided as supplementary files. The ANI values between strain RSS-23^T^ and *T. fusca* DSM 15424^T^ or *T. aquatica* SY21^T^ were 78.10 and 76.11 %, respectively, which were below the 95 % inter-species threshold [30]. The estimated dDDH values of strain RSS-23^T^ to *T. fusca* DSM 15424^T^ and *T. aquatica* SY21^T^ were 25.20 and 20.90 %, respectively, which were below the standard value generally recommended for species differentiation.

The morphology and size of cells grown on R2A agar at 30 °C for 2 days were observed by using transmission electron microscopy (JEM-1400, jeol), and motility was observed with light microscopy. Gram-staining was performed according to Hucker [31]. Growth was measured at temperatures of 16, 20, 30, 37,40, 45, 50, 60, 65 and 70 °C. Anaerobic growth was examined in R2A broth without oxygen, and using l-cysteine (1 g l^−1^) to consume residual oxygen and resazurin (1 mg l^−1^) as a redox indicator. Salt tolerance was investigated by supplementing NaCl concentrations of 0, 1, 2, 3, 4 and 5.0 % (w/v) to R2A broth. The effect of pH (at pH 5.0, 6.0, 7.0, 8.0 and 9.0) was tested in R2A broth using phosphate buffer (for pH 5.0–7.0) and Tris buffer (for pH 8.0–9.0). Catalase activity was determined using a 3 % (v/v) hydrogen peroxide solution. Oxidation of *N*,*N*,*N*′,*N*′-tetramethyl-*p*-phenylenediamine dihydrochloride was used to check oxidase activity [32]. Monosaccharide, disaccharide and polysaccharide utilization was tested with the following compounds and basic inorganic salt at concentration of 1 g 1^−1^: starch, cellulose, chitin, xylan, agar, fructose, galactose, mannose, xylose, arabinose, glucose, lactose, maltose, cellobiose, trehalose, sucrose and sorbose. Bacterial cells grown in R2A were collected and washed three times with basic inorganic salt medium, inoculated at 1 % (v/v), and cultivated for 2 days. Other physiological and biochemical analyses were carried out by using the API ZYM, API 20NE and Biolog GEN III systems, according to the manufacturers’ instructions. Cells of strains 3A5MI-3^T^ and RSS-23^T^ were Gram-stain-negative, aerobic, non-motile and short rod-shaped (Fig. S3). The distinctive physiological characteristics of strains 3A5MI-3^T^ and RSS-23^T^ are listed in Tables 1 and 2, respectively.

**Table 1.** Phenotypic characteristics that differentiate strain 3A5MI-3^T^ from the phylogenetically closely related strains of the genus *Niabella
* Strains: 1, 3A5MI-3^T^; 2, *N. pedocola* JCM 31011^T^ [8]; 3, *N. aquatica* JCM 30952^T^ [2]; 4, *N. aurantiaca* DSM 17617^T^ [6]; 5, *N. ginsengisoli* JCM 15444^T^ [3]; 6, *N. ginsenosidivorans* JCM 18199^T^ [13]; 7, *N. hibiscisoli* KACC 18857^T^ [10]; 8, *N. hirudinis* DSM 25812^T^ [9]; 9, *N. soli* DSM 19437^T^ [12]; 10, ‘* N. terrae *’ JCM 19502^T^ [14]; 11, ‘* N. thaonhiensis *’ JCM 18864^T^ [15]; 12, *N. yanshanensis* KACC 14980^T^ [11]; 13, *N. drilacis* DSM25812^T^ [9]; 14, *N. tibetensis* CCTCC AB 209167^T^ [7]. +, Positive; −, negative; w, weakly positive. The data of strain 3A5MI-3^T^ and *N. pedocola* JCM 31011^T^ were taken from the current study, others were taken from the published literature.

Characteristic	1	2	3	4	5	6	7	8	9	10	11	12	13	14
Source of isolation	Sludge	Soil	Water	Soil	Soil	Compost	Soil	Leeches	Soil	Soil	Soil	Soybean rhizosphere	Leeches	Soil
pH range for growth	5–9	5.5–10.5	5–9	5–8	6–8	4.5–10	5–8	4.5–10.5	5–8	5–8.5	6.5–11	6–10	4.5–10.5	5–8
Temperature range for growth (°C)	15–45	15–40	10–37	10–35	5–35	18–42	4–40	15–30	15–35	15–35	15–37	15–35	15–30	15–34
Catalase/oxidase	−/+	−/w	+/−	+/−	+/−	+/+	+/+	−/+	+/+	+/+	−/+	+/+	−/+	+/+
Gelatin hydrolysis	+	−	−	+	−	−	+	−	−	+	+	+	−	+
Assimilation tests (API 20NE and Biolog GENIII):														
d-Glucose	−	+	+	+	−	+	+	−	+	+	+	+	+	+
d-Mannitol	−	−	−	−	−	−	−	−	+	+	−	−	−	−
l-Fucose	w	−	+	−	+	+	+	−	−	+	−	+	−	−
l-Serine	+	−	+	−	−	−	−	−	−	−	−	−	−	−
l-Alanine	w	−	−	−	−	−	−	+	−	−	−	−	+	−
Enzyme activities (API ZYM):														
Cystine arylamidase	w	+	+	−	+	+	+	+	−	−	w	+	+	+
Trypsin	w	−	−	−	+	−	+	w	−	+	−	+	w	−

**Table 2.** Phenotypic characteristics that differentiate strain RSS-23^T^ from the phylogenetically closely related species of the genus *Thermomonas
* Strains: 1, RSS-23^T^; 2, *T. fusca* DSM 15424^T^ [18]; 3, *T. aquatica* KCTC 62191^T^ [4]; 4, *T. carbonis* KCTC 42013^T^ [17]; 5, *T. brevis* DSM 15422^T^ [18]; 6, *T. haemolytica* DSM 13605^T^ [16]; 7, *T. koreensis* KCTC 12540^T^ [5]; 8, *T. hydrothermalis* DSM 14834^T^ [19]. +, Positive; −, negative; w, weakly positive/sensitive. The data of strain RSS-23^T^ and *T. fusca* DSM 15424^T^ were taken from the current study, others were taken from the published literature.

Characteristic	1	2	3	4	5	6	7	8
Colony colour on R2A agar	Cream	Beige	Cream	Yellow	Beige	Cream	Beige	Cream
Cell shape	Ovoid rod	Rod	Rod	Ovoid rod	Rod	Rod	Rod	Rod
Nitrate reduction	+	+	−	−	−	−	+	+
Optimum growth temperature (°C)	30	28–37	28	28	28–37	37–50	37	50
Motility	−	+	−	−	+	+	+	−
Aesculin hydrolysis	+	−	−	+	+	−	+	+
Gelatin hydrolysis	+	+	−	−	+	−	+	+
Assimilation tests (API 20NE and Biolog):								
d-Glucose	+	−	+	−	+	−	+	+
Maltose	+	−	+	w	+	−	+	+
d-Mannitol	−	−	+	+	−	−	−	−
*N*-Acetylglucosamine	+	−	w	+	+	−	−	−
d-Mannose	−	−	+	−	+	−	−	−
l-Proline	+	+	+	+	+	−	+	+
Enzyme activities (API ZYM):								
Trypsin	+	+	+	w	+	−	−	−
β-Glucosidase	+	−	+	−	−	−	+	+
Cystine arylamidase	+	+	+	w	+	w	+	−
Chymotrypsin	+	+	−	w	−	+	−	+
Lipase (C14)	−	−	−	−	−	+	−	+

The cellular fatty acids were determined using cells grown on R2A agar for 2 days at 30 °C with the standard midi protocol (Sherlock Microbial Identification System, version 6.0B) and analysed with a gas chromatograph (GC6890, Agilent) [33]. The extraction, purification and analysis of quinones were carried out according to the methods of Collins *et al*. [34]. The total lipids were extracted using chloroform and methanol, followed by thin-plate biphasic chromatography to identify each lipid component [35].

Strain 3A5MI-3^T^ contained aliphatic saturated fatty acid (iso-C_15 : 0_) as its predominant cellular fatty acid that accounted for about half of total fatty acids, followed by iso-C_15 : 1_ ω6*c* and/or iso-C_15 : 1_ ω7*c*, iso-C_17 : 0_ 3-OH and summed feature 3 (C_16 : 1_ ω7*c*/C_16 : 1_ ω6*c*). The composition of the fatty acids is shown in Table 3. The major cytoquinone was determined to be MK-7, as previously reported for described members of the genus *Niabella *. The polar lipid profile of strain 3A5MI-3^T^ contained phosphatidylethanolamine and three unknown lipids (Fig. S4a).

**Table 3.** Cellular fatty acid composition (% of total) of strain 3A5MI-3^T^ and closely related species of the genus *Niabella
* Strains: 1, 3A5MI-3^T^; 2, *N. pedocola* JCM 31011^T^ [8]; 3, *N. aquatica* JCM 30952^T^ [2]; 4, *N. aurantiaca* DSM 17617^T^ [6]; 5, *N. ginsengisoli* JCM 15444^T^ [3]; 6, *N. ginsenosidivorans* JCM 18199^T^ [13]; 7, *N. hibiscisoli* KACC 18857^T^ [10]; 8, *N. hirudinis* DSM 25812^T^ [9]; 9, *N. soli* DSM 19437^T^ [12]; 10, ‘* N. terrae *’ JCM 19502^T^ [14]; 11, ‘* N. thaonhiensis *’ JCM 18864^T^ [15]; 12, *N. yanshanensis* KACC 14980^T^ [11] ; 13， *N. drilacis* DSM25812^T^ [9]; 14, *N. tibetensis* CCTCC AB 209167^T^ [7]. −, Not detected or <0.1 %. The data of strain 3A5MI-3^T^ and *N. pedocola* JCM 31011^T^ were taken from the current study, others were taken from the published literature.

Fatty acid	1	2	3	4	5	6	7	8	9	10	11	12	13	14
Saturated:														
iso-C_15 : 0_	40.6	40.9	40.4	33.7	32.8	42.0	27.5	52.4	29.2	38.9	36.7	39.4	40.8	41.3
anteiso-C_15 : 0_	1.9	1.2	0.9	1.6	–	0.7	0.6	–	1.2	3.8	0.9	–	0.7	0.9
C_16 : 0_	5.5	5.9	2.2	3.5	7.9	2.1	13.3	2.9	6.8	2.6	3.6	4.3	3.2	3.4
Unsaturated:														
iso-C_15 : 1_ G*	19.0	32.2	23.4	22.3	36.4	15.9	20.3	16.6	18.4	20.3	20.8	30.5	20.5	14.9
anteiso-C_15 : 1_	1.1	1.0	–	–	–	–	–	–	–	–	–	–	–	–
Hydroxy:														
iso-C_15 : 0_ 3OH	1.7	2.1	2.8	2.9	3.7	2.9	3.0	3.8	2.2	3.0	2.6	3.2	–	2.4
iso-C_16 : 0_ 3OH	1.1	–	–	–	–	0.7	–	–	–	2.2	–	–	–	0.7
C_16 : 0_ 3OH	2.4	–	1.8	2.4	–	1.2	1.8	1.9	2.2	1.8	2.9	1.4	3.5	3.3
iso-C_17 : 0_ 3OH	11.4	7.6	13.9	15.5	9.5	16.4	10.7	9.7	11.8	12.9	9.1	7.7	9.2	–
Summed feature 3†	7.1	5.2	12.5	10.6	3.5	14.4	8.8	5.2	11.1	5.3	15	7.8	10.7	16.0

*iso-C_15 : 1_ G should correspond to either iso-C_15 : 1_ ω6*c* and/or iso-C_15 : 1_ ω7*c*. The double bond position indicated by a capital letter is unknown.

†Summed features are fatty acids that cannot be resolved reliably from another fatty acid using the chromatographic conditions chosen. The midi system groups these fatty acids together as one feature with a single percentage of the total. Summed feature 3 comprised C_16 : 1 _ω7*c*/C_16 : 1 _ω6*c*.

Strain RSS-23^T^ also contained aliphatic saturated fatty acid (iso-C_15 : 0_) as its predominant cellular fatty acid, which accounted for about one third of total fatty acids. The fatty acid profiles are shown in Table 4. The major isoprenoid quinone was determined to be Q-8, as previously reported for members of the genus *Thermomonas *. The polar lipid profile contained diphosphatidylglycerol, phosphatidylglycerol, phosphatidylethanolamine, two unknown phospholipids and an unknown lipid (Fig. S4b).

**Table 4.** Cellular fatty acid composition (% of total) of strain RSS-23^T^ and closely related species of the genus *Thermomonas
* Strains: 1, RSS-23^T^; 2, *T. fusca* DSM 15424^T^ [18]; 3, *T. aquatica* KCTC 62191^T^ [4]; 4, *T. carbonis* KCTC 42013^T^ [17]; 5, *T. brevis* DSM 15422^T^ [18]; 6, *T. haemolytica* DSM 13605 [16]^T^ [16]; 7, *T. koreensis* KCTC 12540^T^ [5]; 8, *T. hydrothermalis* DSM 14834^T^ [19]. −, Not detected or <0.1 %. The data of strain RSS-23^T^ and *T. fusca* DSM 15424^T^ were taken from the current study, others were taken from the published literature.

Fatty acid	1	2	3	4	5	6	7	8
Saturated:								
iso-C_11 : 0_	4.9	4.3	7.8	8.8	11.0	9.7	8.7	9.5
iso-C_13 : 0_	1.3	2.5	–	–	–	–	2.9	–
C_14 : 0_	4.8	3.4	4.3	3.2	1.3	–	1.7	–
iso-C_14 : 0_	4.3	13.8	–	1.4	1.7	2.9	2.6	–
iso-C_15 : 0_	31.1	21.9	27.9	35.8	44.3	48.7	47.4	52.3
anteiso-C_15 : 0_	2.9	1.8	1.9	–	1.1	–	–	–
C_16 : 0_	4.2	1.9	13.4	6.0	1.9	1.4	–	2.0
iso-C_16 : 0_	5.0	11.1	2.3	2.7	3.0	10.4	1.5	1.2
iso-C_17 : 0_	1.0	–	1.7	1.2	1.7	3.9	–	7.6
Unsaturated:								
iso-C_15 : 1_ F*	5.7	3.0	1.3	3.1	1.8	1.0	8.3	1.2
iso-C_17 : 1_ ω9*c*	15.7	12.5	–	7.9	17.6	8.0	16.1	13.8
C_16 : 1_ ω9*c*	1.5	1.0	–	–	–	–	–	–
Hydroxy:								
iso-C_11 : 0_ 3-OH	6.3	5.9	10.3	7.8	8.6	10.6	5.9	8.0
Summed feature 3†	7.9	10.3	–	17.5	2.2	–	1.5	–

*iso-C_15 : 1_ F should correspond to either iso-C_15 : 1_ ω6*c* and/or iso-C_15 : 1_ ω5*c*. The double bond position is presumptive [36].

†Summed features are fatty acids that cannot be resolved reliably from another fatty acid using the chromatographic conditions chosen. The midi system groups these fatty acids together as one feature with a single percentage of the total. Summed feature 3 comprised C_16 : 1 _ω7*c*/C_16 : 1 _ω6*c*.

Based on the phylogenetic and phenotypic characteristics, we conclude that strains 3A5MI-3^T^ and RSS-23^T^ represent novel species of the genera *Niabella* and *Thermomonas *, respectively, for which the names *Niabella beijingensis* sp. nov. and *Thermomonas beijingensis* sp. nov. are proposed.

## Description of *Niabella beijingensis* sp. nov.

*Niabella beijingensis* (bei.jing.en’sis. N.L. fem. adj. *beijingensis*, pertaining to Beijing, the geographical origin of the type strain).

Cells are Gram-stain-negative, strictly aerobic, non-motile and short rod-shaped (Fig. S3a). Colonies are convex, circular, smooth and edges were regular after incubation on R2A broth for 2 days at 30 °C. The strain can grow in a wide range of temperature (16–45 °C) and pH (5.0–9.0), and the range of NaCl tolerance is 0–2 % (w/v) NaCl. Optimal growth at 30 °C, pH 7.0 and with 1 % NaCl (w/v). Oxidase and catalase activities are positive and negative, respectively. According to the API 20NE system, nitrate cannot be reduced to nitrite. Positive for aesculin ferric citrate, gelatin, 4-nitrophenyl-β-d-galactopyranoside d-glucose, l-arabinose, d-mannose, *N*-acetyl-glucosamine and maltose, and negative for l-tryptophan, l-arginine, urea, d-mannitol, potassium gluconate, capric acid, adipic acid, malic acid, trisodium citrate and phenylacetic acid (API 20NE). The following enzyme activities are positive: alkaline phosphatase, leucine arylamidase, valine arylamidase, acid phosphatase, naphthol-AS-BI-phosphohydrolase, α-galactosidase, β-galactosidase, α-glucosidase, β-glucosidase, *N*-acetyl-β-glucosaminidase, α-mannosidase and α-fucosidase. Esterase lipase (C8), cystine arylamidase and trypsin are weak; and esterase (C4), lipase (C14), α-chymotrypsin and β-glucuronidase are negative (API ZYM). Carbon substrates utilized include gentiobiose, sucrose, stachyose, maltose, trehalose, cellobiose, turanose, raffinose, lactose, melibiose, d-mannose, d-fructose, d-galactose, l-rhamnose, glycy-l-proline, l-aspartic acid, l-glutamic acid, l-serine, dextrin, pectin, Tween 40, acetoacetic acid, acetic acid, l-lactic acid, *N*-acetyl neuraminic acid, d-galacturonic acid, d-glucuronic acid, l-galactonic acid lactone, methyl β-d-glucoside, d-salicin, *N*-acetyl-d-glucosamine, *N*-acetyl-d-galactosamine, glucuronamide, gelatin, d-arabinose, d-sorbose, starch and agar. Weak utilization of l-fucose, d-glucose-6-PO4, l-alanine, l-histidine, d-gluconic acid, citric acid, glycerol, *N*-acetyl-β-d-mannosamine, methyl pyruvate, xylan and cellulose. No utilization of 3-methyl glucose, d-fucose, inosine, d-sorbitol, d-mannitol, d-arabitol, *myo*-inositol, d-fructose-6-PO4, d-aspartic acid, d-serine, l-arginine, l-pyroglutamic acid, mucic acid, quinic acid, d-saccharic acid, *p*-hydroxy-phenylacetic acid, d-lactic acid methyl ester, α-keto-glutaric acid, d-malic acid, l-malic acid, bromo-succinic acid, γ-amino-butryric acid, β-hydroxy-d,l-butyric acid, α-keto-butyric acid, propionic acid, formic acid and chitin (Biolog GENⅢ and cultivation examinations). The predominant cellular fatty acids are iso-C_15 : 0_, iso-C_15 : 1_ ω6*c* and/or iso-C_15 : 1_ ω7*c*, iso-C_17 : 0_ 3-OH and summed feature 3 (C_16 : 1_ ω7*c*/C_16 : 1_ ω6*c*). Contains cytoquinone MK-7 as respiratory quinone. The polar lipid profile contains phosphatidylethanolamine and three unknown lipids. The G+C content of the type strain is 47.07 mol%.

The type strain, 3A5MI-3^T^ (=CGMCC 1.17737^T^=KCTC 82817^T^), was isolated from constructed wetland sludge from Beijing, PR China.

## Description of *Thermomonas beijingensis* sp. nov.

*Thermomonas beijingensis* (bei.jing.en’sis. n.l. fem. adj. *beijingensis*, pertaining to Beijing, the geographical origin of the type strain).

Cells are Gram-stain-negative, aerobic, non-motile and rod-shaped. Colonies are convex, circular, smooth and edges are regular after incubation on R2A broth for 2 days at 30 °C. The strain can grow in a wide range of temperature (16–45 °C) and pH (5.0–9.0). The range of NaCl tolerance is 0–1 % NaCl (w/v). Optimal growth at 30 °C, pH 7.0 and with 1 % NaCl (w/v). Oxidase and catalase activities are positive. According to the API 20NE system, nitrate can be reduced to nitrite. Positive for aesculin ferric citrate, gelatine, d-glucose, *N*-acetyl-glucosamine and maltose, and negative for l-tryptophan, l-arginine, urea, 4-nitrophenyl-β-d-galactopyranoside, l-arabinose, d-mannose, d-mannitol, potassium gluconate, caprate, adipate, malate, citrate and phenylacetate (API 20NE). The following enzyme activities of alkaline phosphatase, leucine arylamidase, valine arylamidase, acid phosphatase, naphthol-AS-BI-phosphohydrolase, α-glucosidase, β-glucosidase, *N*-acetyl-β-glucosaminidase, esterase lipase (C8), cystine arylamidase, trypsin, esterase (C4) and α-chymotrypsin are positive; α-mannosidase. α-fucosidase, lipase (C14), α-galactosidase, β-galactosidase and β-glucuronidase are negative (API ZYM). Carbon sources utilized include dextrin, maltose, cellobiose, gentiobiose, *N*-acetyl-d-glucosamine, *N*-acetyl-d-galactosamine, gelatin, glycy-l-proline, l-alanine, l-arginine, l-aspartic acid, l-glutamic acid, l-serine, methyl pyruvate, Tween 40, α-hydroxy-butyric acid, β-hydroxy-d,l-butyric acid, α-keto-butyric acid, acetoacetic acid, propionic acid, acetic acid, d-arabinose, d-sorbose, starch, xylan and agar. Weak utilization of turanose, *N*-acetyl-β-d-mannosamine, d-fructose, d-glucose-6-PO_4_, d-aspartic acid and pectin. No growth occurs on trehalose, sucrose, stachyose, raffinose, lactose, melibiose, methyl β-d-glucoside, d-salicin, *N*-acetyl neuraminic acid, α-d-glucose, d-mannose, d-galactose, 3-methyl glucose, d-fucose, l-fucose, l-rhamnose, inosine, d-sorbitol, d-mannitol, d-arabitol, *myo*-inositol, glycerol, d-serine, l-histidine, l-pyroglutamic acid, d-galacturonic acid, l-galactonic acid lactone, d-gluconic acid, d-glucuronic acid, glucuronamide, mucic acid, quinic acid, d-saccharic acid, *p*-hydroxy-phenylacetic acid, d-lactic acid methyl ester, l-lactic acid, citric acid, α-keto-glutaric acid, d-malic acid, l-malic acid, bromo-succinic acid, γ-amino-butryric acid, formic acid, cellulose and chitin (Biolog GENⅢ and cultivation examinations). The predominant cellular fatty acids are iso-C_15 : 0_, iso-C_17 : 1_ ω9*c*, iso-C_11 : 0_ 3-OH and summed feature 3 (C_16 : 1_ ω7*c*/C_16 : 1_ ω6*c*). The major isoprenoid quinone is Q-8. The polar lipid profile contains diphosphatidylglycerol, phosphatidylglycerol, phosphatidylethanolamine, two unknown phospholipids and an unknown lipid.

The type strain, RSS-23^T^ (=CGMCC 1.17738^T^=KCTC 82820^T^), was isolated from soil of the Dragon-shaped Wetland System in Beijing Olympic Park, PR China. The DNA G+C content of the type strain is 61.21 mol%.
